# Use of ketamine in Super Refractory Status Epilepticus: a systematic review

**DOI:** 10.1186/s42466-024-00322-7

**Published:** 2024-06-27

**Authors:** Aayush Adhikari, Sushil Kumar Yadav, Gaurav Nepal, Roshan Aryal, Pratik Baral, Peter Neupane, Aadesh Paudel, Barsha Pantha, Sulav Acharya, Gentle Sunder Shrestha, Ramesh Khadayat

**Affiliations:** 1 Manang Hospital, 33500 Chame, Manang, Nepal; 2 Intern, Institute of Medicine, Tribhuvan University Teaching Hospital, 44600 Kathmandu, Nepal; 3 Jibjibe Primary Health Care Centre, 45003 Dhaibung, Rasuwa, Nepal; 4 National Medical College, 44300 Birgunj, Nepal; 5 Post Graduate Institute of Medical Education and Research, 160012 Chandigarh, India; 6 Department of Critical Care Medicine, Tribhuvan University Teaching Hospital, 44600 Maharajgunj, Kathmandu, Nepal

**Keywords:** SRSE, Convulsive Status Epilepticus (CSE), Non-convulsive Status Epilepticus (NCSE), NMDA agonist, Neuroprotection, STESS score, Burst suppression

## Abstract

**Objective:**

This review specifically investigates ketamine’s role in SRSE management.

**Methods:**

PubMed, EMBASE, and Google Scholar databases were searched from inception to May 1st, 2023, for English-language literature. Inclusion criteria encompassed studies on SRSE in humans of all ages and genders treated with ketamine.

**Results:**

In this systematic review encompassing 19 studies with 336 participants, age ranged from 9 months to 86 years. Infections, anoxia, and metabolic issues emerged as the common causes of SRSE, while some cases had unknown origins, termed as NORSE (New Onset RSE) or FIRESs (Febrile Infection-Related Epilepsy Syndrome). Most studies categorized SRSE cases into convulsive (*N* = 105) and non-convulsive (*N* = 197). Ketamine was used after failed antiepileptics and anesthetics in 17 studies, while in others, it was a first or second line of treatment. Dosages varied from 0.5 mg/kg (bolus) and 0.2-15 mg/kg/hour (maintenance) in adults and 1-3 mg/kg (bolus) and 0.5-3 mg/kg/hour (maintenance) in pediatrics, lasting one to 30 days. Ketamine was concurrently used with other drugs in 40–100% of cases, most frequently propofol and midazolam. Seizure resolution rate varied from 53.3 to 91% and 40–100% in larger (*N* = 42–68) and smaller case series (*N* = 5–20) respectively. Seizure resolution occurred in every case of case report except in one in which the patient died. Burst suppression in EEG was reported in 12 patients from two case series and two case reports. Recurrence was reported in 11 patients from five studies. The reported all-cause mortality varied from 38.8 to 59.5% and 0-36.4% in larger and smaller case series., unrelated directly to ketamine dosage or duration.

**Significance:**

Ketamine demonstrates safety and effectiveness in SRSE, offering advantages over GABAergic drugs by acting on NMDA receptors, providing neuroprotection, and reducing vasopressor requirement.

**Supplementary Information:**

The online version contains supplementary material available at 10.1186/s42466-024-00322-7.

## Key points

- 1. Infection, anoxia and metabolic cause were the most commonly reported etiology
- 2. Ketamine showed better efficacy when administered earlier
- 3. Ketamine infusion significantly resulted in decreased vasopressor requirement

**Supplementary Information:**

The online version contains supplementary material available at 10.1186/s42466-024-00322-7.

## Introduction

Status Epilepticus (SE) is a life-threatening medical emergency with high morbidity and mortality and results from either the failure of the mechanisms responsible for seizure termination or from the initiation of mechanisms that lead to abnormally prolonged seizures. The initial treatment is a rapidly-acting benzodiazepine, which is followed by intravenous anti-seizure medications (ASMs) in loading and maintenance doses. When seizure fails to control despite first- and second- line treatments, we call it Refractory Status Epilepticus (RSE) and occurs in approximately 20% of patients with status epilepticus [1–3]. Studies have shown that longer seizures are less likely to stop spontaneously and are also less responsive to anti-seizure medications [4, 5]. This condition requires continuous infusion of IV anesthetic agents. However, there are cases where seizure continues or recurs 24 h or more after intravenous infusion of anesthetic therapy which is labeled as Super Refractory Status Epilepticus (SRSE). SRSE also includes cases when SE recurs on reduction or withdrawal of anesthetic agents [5, 6].

Ketamine is a noncompetitive antagonist of the NMDA-glutamate receptor and it stands out as a promising therapy in RSE and SRSE when GABA-ergic agents have failed. Also its sympathomimetic action acts as a safeguard against cardiac depression when the use of other conventional intravenous anesthetic agents would be limited by hypotension [7]. In addition to these effects, Ketamine also has a potential to prevent glutamate-mediated neurotoxicity and respiratory depression [8].

Studies demonstrating the clinical efficacy of ketamine on patients with prolonged RSE is scarce. There are a few retrospective case studies supporting the use of Ketamine in prolonged SE/SRSE but prospective randomized controlled trials supporting its use are still lacking [9]. Ketamine is administered only when conventional anesthetics have failed; however, there are newer notions regarding its earlier administration owing to its potential efficacy and good safety profile [10, 11]. Herein, we performed a systematic review of the literature on the use of Ketamine in treating SRSE in pediatric and adult populations.

## Methodology

This systematic review is reported according to the PRISMA (Preferred Reporting Items for Systematic Reviews and Meta-Analyses) statement 2020, following the PRISMA checklist and flow diagram for manuscript format development [12]. The primary focus of our study was to investigate the effectiveness of Ketamine in treating Super-Refractory Status Epilepticus (SRSE) across both pediatric and adult populations. We sought to analyze various etiology/semiology of SRSE, latency/dose/duration/adverse effects of ketamine administration, and outcomes (including seizure resolution rate, EEG features post ketamine administration, recurrence of SRSE, functional outcome, and mortality rates). Then, we drafted our inclusion and exclusion criteria.

### Study inclusion and exclusion criteria

The inclusion criteria for the studies encompassed the following requirements: (1) Individuals diagnosed with Super-Refractory Status Epilepticus (SRSE), based on clinical features, EEG findings, or both. (2) Participants spanning both pediatric and adult age groups. (3) Studies delineating the use of ketamine, including its dose, route of administration, and duration of therapy for SRSE. (4) Documentation of seizure resolution rate with or without post-Ketamine EEG characteristics, recurrence rates, functional outcomes, mortality rates, adverse effects, and hemodynamic effects.

The exclusion criteria were as follows: (1) Animal or in vitro studies. (2) Insufficient data availability. (3) Duplicate articles. (4) Reviews or meta-analyses. (5) Studies not published in English.

### Search methods and study selection

PubMed, EMBASE, and Google Scholar databases were searched from inception to May 1, 2023 for English language literature. Boolean logic was used for conducting a database search, and Boolean search operators “AND” and “OR” were used to link search terms: ‘ketamine’, ‘Refractory Status Epilepticus’, ‘Super Refractory Status Epilepticus ‘, ‘RSE’, ‘SRSE’ and ‘prolonged RSE’. The detailed PubMed search strategy was as follows: “Ketamine“[MeSH Terms] AND (“Refractory Status Epilepticus“[All Fields] OR “Super Refractory Status Epilepticus“[All Fields] OR “RSE“[All Fields] OR “SRSE“[All Fields] OR “Prolonged Refractory Status Epilepticus“[All Fields]). We also searched the reference list of each included study to identify other potential material of interest. All shortlisted studies were then imported to the Mendeley, and duplicates were removed appropriately. Papers were initially reviewed by title, keywords, and abstract by two reviewers (SKY and PB) independently and subsequently verified with a third reviewer (AA). Articles after the initial screen were subsequently reviewed in full by two reviewers (SKY and PB). We resolved the final study selection differences between the two primary reviewers (SKY and PB) by the discussion with a third reviewer (AA). An overall evaluation for potential overlap of the population was conducted based on authorship, hospital setting, and recruitment period. In cases of overlap, studies of higher quality or larger sample sizes were planned to be included. Quality assessment was done by checking the clarity of study designs/objectives, study population, presentation of results, analysis/statistics, bias/confounding minimization, outcome measures, relevance of the study findings, ethical considerations, thoroughness of reporting, depth of discussion/conclusion, and generalizability.

### Data extraction

Two independent authors (SKY and PB) rigorously reviewed and selected studies for systematic review which met our inclusion criteria and extracted the precise information on different headings under four tables depicting baseline features (Author/ Year published, Study site, Study design, Study period, Total participants/total SE episodes/age group, Sex, Etiology, and SE Semiology) in Table 1, parameters of ketamine use (Latency to KE, Previous ASMs/Anesthetics, KE dose, KE duration, Proportion of concurrent drug receiver/ drugs) in Table 2, clinical outcomes (Seizure resolution rate /Resolution time post ketamine, EEG features post-ketamine, Recurrence of SE during hospitalization/ follow up, Functional outcome at discharge, Adverse effects, All-cause mortality) in Table 3, and hemodynamic effect in Table 4. Microsoft Excel 2013 (Microsoft Corp, Redmond, USA) was used for data extraction.

**Table 1:** Baseline characteristics of studies included in this systematic review

Author/ Year published	Study site	Study design	Study period	Total participants/total SE episodes/age group	Median Age (Range)	Sex(F/M)	Etiology (N)	SE Semiology
**Larger case series**								
Gaspard 2013	North America and Europe	Retrospective	1999–2012	58/60/All (12 *P* + 46 A)	24 y (7 m-74 y)	30/30	I-CNS (4), I-systemic (1) Anti-NMDARE (2), SAH (2), IS (2), TBI (1), PRES (1), PAE (7), NORSE (34), Remote symptomatic (6)	19 CSE + 41 NCSE
Sabharwal 2015	USA	Retrospective	2012–2015	67/67/All	62 y (8 y-85 y)	49/18	PAE (13), IS (4), HS (3), M/Tx (18), I-CNS (5), I-systemic (5), AI (3), T (3), G (2), NORSE (11)	NCSE
Hofler 2016	Austria	Retrospective	2011–2015	42 (3 RSE + 39 SRSE)/42/A	67 y (59.3 y-72 y) ^€^	20/22	PAE (14), IS/HS (7), I-CNS (4), T (3), PHS (7), NORSE (7)	14 CSE + 28 NCSE
Alkhachroum2020	USA	Retrospective	2009–2018	68/68/A	53 ± 19 y ^£^	46/22	CA (18), NORSE (12), IS/HS/SAH (11), I (8), E (6), O (13)	18 CSE + 50 NCSE
**Smaller case series**								
Mewasingh 2003	Belgium	Case Series	NR*	5/5/P*	4 y (4 y– 7 y)	3/2	LGS (2), PME (1), MAE (1), ABPE (1)	NCSE
Rosati 2012	Italy	Case Series	2009–2011	9 (1 RSE and 8 SRSE) /11/P	4 y 8 m (1 y 4 m– 10 y 5 m)	5(7 cases)/4	MELAS (1), RS (1), SPE (2), FIRES (2), U (5)	CSE
Synowiec 2013	USA	Case Series	2003–2011	11/11/A	53 y (22 y– 82 y	4/7	I (7), Low ASM (3), M (1)	6 NCSE + 5 CSE
Basha 2015	USA	Case Series	2011–2013	11/11/A	56 y (33 y– 68 y)	6/5	HS (2), PAE (1), Encephalomalacia and I (1), MNC (1), mucocele (1), MBL (1), Rt. medial temporal sclerosis, AI (1), U (2)	1 NCSE + 10 CSE
Liaqat 2018	Pakistan	Case Series	Jan 2014-Dec 2014	20 (2 RSE + 18 SRSE)/20/A	52.8 ± 18.32 ^£^	9/11	U (11), PAE (2), I-CNS (1), SAH (1), IS (1), TBI (1), E (3)	18 CSE + 2 NCSE
Wang 2020	China	Retrospective	2016–2018	18 (7 RSE + 11 SRSE)/18/P	6 y 8 m (9 m -16 y)	9/9	FIRES (8), I-CNS (7), E (2), SSADD (1)	NR
Dericioglu 2020	Turkey	Retrospective	2009–2019	7/7/A	66 y (44 y– 86 y)	3/4	I-CNS (3), IS/HS (2), HIE (1), I-CNS + HIE (1)	NCSE
Caranzano 2022	Switzerland	Prospective registry	2006-21	11/11/A	46 y (20 y– 78 y)	5/6	NORSE/FIRES (4), AI (1), MNC (1), SAH (1), I-CNS (2), CA (1), M (1)	8 NCSE + 1 CSE + 2 Partial Complex
**Case reports**								
Hsieh 2010	Taiwan	Case report	NR	1/1/A	23 y	0/1	U (1)	CSE
Shrestha 2015	Nepal	Two case reports	NR	2/2/A	23 y and 30 y	2/0	U (2)	CSE
Mutkule 2018	India	Case Report	NR	1/1/P	18 y	0/1	Synthetic Marijuana abuse (1)	CSE
Santoro 2019	USA	3 case reports	NA	3/3/All	3 y, 19 y, and 54 y	2/1	Anti-NMDARE (3)	CSE
Samanta 2020	USA	2 Case Reports	NR	2/2/P	3 y and 6 y	1/1	AHC (2)	CSE
Meenakshi-Sundaram 2020	India	Case report	NR	1/1/P	14 y	0/1	FIRES (1)	CSE
Manganotti 2021	Italy	Case report	NR	1/1/A	23 y	0/1	TBI (1)	CSE

*Abbreviations* SE-status epilepticus, NCSE-Non-Convulsive Status Epilepticus, CSE-convulsive Status Epilepticus, A-Adult, P-Pediatric (≤ 18 Years), h-hours, d-days, m-months, y-years, RSE-Refractory Status Epilepticus, SRSE- Super Refractory Status Epilepticus, I-infection (CNS and/or systemic), AI-autoimmune, Anti-NMDARE- anti-NMDAR encephalitis, SAH-subarachnoid hemorrhage, IS-ischemic stroke, HS-hemorrhagic stroke, TBI-traumatic brain injury, PRES-posterior reversible encephalopathy syndrome, PAE- post anoxic encephalopathy, T-tumor, G-genetic, U-unknown, NORSE- new onset RSE of unknown origin, M-metabolic, Tx-toxic, PHS-previous history of seizure, E-epilepsy, LGS-Lennox-Gastaut Syndrome, PME-Progressive Myoclonic Epilepsy, MAE-Myoclonic-astatic Epilepsy, ABPE-Atypical Benign Partial Epilepsy, MELAS-Mitochondrial Encephalomyopathy, RS-Rett syndrome, SPE-symptomatic partial epilepsy, FIRES- Febrile illness related epilepsy syndrome, MNC- medication non-compliance, MBL- metastatic brain lesions, SSADD-Succinate semialdehyde dehydrogenase deficiency, HIE-hypoxic ischemic encephalopathy, CA-cerebral abscess, AHC-alternating hemiplegia of childhood, O-others. ^€^ Interquartile range; ^£^ Mean ± S.D

**Table 2:** Ketamine administration parameters

Author/ Year published	Latency to KE, median (range)	Previous ASMs/Anesthetics	KE dose, Median (Range)	KE duration, Median (Range)	Proportion of concurrent drug receiver/ drugs
**Larger Case Series**					
Gaspard 2013	9 d (6 h– 122 d)	PR, MDZ, PentB and TP	LD- 1.5 mg/kg (maximum 5 mg/kg) MD- 2.75 mg/kg/h (0.05–10 mg/kg/h)	4 d (6 h to 27 d)	100%/ PentB, TP, MDZ and PR (2–12 drugs in every case) ^€^
Sabharwal 2015	NR	PR (in 61 patients) ^£^	MD- NR (1.5–10.5 mg/kg/h)	5.97 d (1 d– 29 d)	100%/ PR (25–140 mcg/kg/min)
Hofler 2016	3 d (2 d– 6.8 d) ^€^	A median of two anesthetics and three antiepileptic drugs ^£^	LD- 200 mg (200 mg to 250 mg) ^α^ MD- 2.39 mg/kg/h (1.52–3.02 mg/kg/h)	4 d (2 d– 6.8 d)	40%/PR
Alkhachroum2020	2 d (1 d − 4.5 d) ^¥^	LEV, PHT, LCM, VPA, CLB, PB, GBP	MD- 2.2 mg/kg/h (0.2 mg/kg/h– 10 mg/kg/h)	2 d (1 d– 4 d)	100%/MDZ (100%), PR (53%), and PentB (14.7%)
**Smaller Case Series**					
Mewasingh 2003	28 d (14 d– 70 d)	2 patients- MDZ and LZP, 3 patients - KE as 1st line of therapy.	1.5 mg/kg/day (orally in two divided dose)	5 d in all patients	100%/ with maintenance ASMs (VPA, LTG, ETM, CZP, FBM, CLB, TPM)
Rosati 2012	6 d (2 d-26 d)	MDZ, TPH, and PR (*N* = 9,5 and 4 respectively)	LD- NR (2–3 mg/kg, two boluses 5 min apart) MD- 2.4 mg/kg/h (0.6–3.6 mg/kg/h)	6 d (3 d– 17 d)	100%/ MDZ ^$^, RUF, CZP, PB, STP, CLB, TPM, LZP, FBM, VPA, PHT, PR
Synowiec 2013	5 d (1 d– 11 d)	PR, LZP, PentB, MDZ, MDZ + PR (*N* = 7,1,1,1, and 1 respectively)	LD- 1 mg/kg (*N* = 3) and 2 mg/kg (*N* = 7) MD- 1.3 mg/kg/h^#^ (0.45–2.1 mg/kg/h)	5 d (4 d − 28 d)	100%/ PR, LZP, PentB, PB, VPA, PHT, CBZ, GBP, TPM, LEV, LTG, DZP,
Basha 2015	4 d (16 h– 11 d)	≥ 1 IV anesthetics + ASMs (1–5 agents)	LD- 1.1 mg/kg, 4.3 mg/kg, and 4 mg/kg (*N* = 2,1, and 1 respectively) MD- 4 mg/kg/h (1–5 mg/kg/h)	3.5 d (2 d– 26 d)	100%/ MDZ, PR, and PentB
Liaqat 2018	NR	MDZ, PHT and LEV.	LD- 5 mg/kg (*N* = 20) MD- 5 mg/kg/h (*N* = 20)	NR	60%/ TP and PR ^µ^
Wang 2020	4 d (1.8 d − 6.3 d)	MDZ and PR in most cases^£^	LD- 1.5 mg/kg (0.3–1.6 mg/kg, *N* = 7) MD- 2.2 mg/kg/h (1.2–5.3 mg/kg/h, *N* = 18)	4 d (2 d– 11 d)	100%/ MDZ, PR, VPA, LEV, PB, Oxcarbazepine, CZP, TPM, NZP
Dericioglu 2020	6 d (4 d– 19 d) ^¥^	LEV, CZP, TPM, oxcarbazepine, LCM, PHT, MDZ, PR	LD- NR (0.5-2 mg/kg, *N* = 5) MD- NR (1–5 mg/kg/h)	8 d (3 d − 24 d)	86%/ MDZ (*N* = 4), PR (*N* = 2), TP (*N* = 1)
Caranzano 2022	4 d (2 d– 20 d)	LEV, PR LCM, MDZ TP, TPM, PB, CBZ, FosPHT PGe, PP, CZP, CLB,	MD- 5 mg/kg/h (2.5–15 mg/kg/h)	2 d (1 d– 16 d)	100%/ MDZ, PR, CZP, PHT, LEV, TPM, PG, PentB, VPA, LCM,
**Case Report**					
Hsieh 2010	58 d	DZP, VPA, MDZ, LEV, PHT, TPM, PR, TP	LD- 0.5 mg/kg MD- 0.38 mg/kg/h	5 days	100%/ MDZ
Shrestha 2015	36 h and 42 h.	1st case (LZP bolus, loading dose of PHT, Sodium VPA, LEV, PB, MDZ), 2nd case (MDZ bolus, PHT loading dose, Sodium VPA + LEV + CLB-Maintenance ASMS)	LD- 1 mg/kg (50 mg and 35 mg) MD- 2 mg/kg/h (100 mg/h and 70 mg/h)	3 d and 2 d	100%/MDZ
Mutkule 2018	4 d	MDZ, LEV, LCM, PHT, CLB, VPA, PB, TPM, TP.	LD- 1 mg/kg MD- 2 mg/kg/h	7 d	None
Santoro 2019	9 d, 4 d, 32 d	MDZ, LEV, VPA, PHT, LZP, CLB, PB, LCM, DZP, GBP, ketogenic diet.	LD- 40 mg, 50 mg, and 40 mg MD- 3 mg/kg/h (*N* = 3)	21 d, 21 d, and 14 d	66%/PB, PHT or other ASMs
Samanta 2020	36 h and 30 h	1st case (DZP, LZP, LEV, MDZ, CZP, VPA, oxcarbazepine, TPM), 2nd case (MDZ, IV LZP, fosPHT, LEV, LCM, PR)	LD- 2 mg/kg in both MD- 0.5–2.5 and 3 mg/kg/h	2 days and 1.5 days	None
Meenakshi-Sundaram 2020	3 d	IV LEV, LCM, MDZ infusion, TP, TPM, PB, CBZ, FosPHT, PP, CZP, CLB, Magnesium, Ketogenic diet	LD- 3 mg/kg MD- 3 mg/kg/h	30 d	100%/Multiple ASMs and anesthetics
Manganotti 2021	12 d	IV PR, LEV, infusion of MDZ, VPA, PHT, LCM,	LD- 3 mg/kg MD- 10 mg/kg/h	3 d	100%/PP

*Abbreviations* IV- Intravenous; CBZ - carbamazepine; CLB - clobazam; CZP - clonazepam; ETM - ethosuximide; FBM - felbamate; KE - ketamine; LEV - levetiracetam; LTG - lamotrigine; LZP - lorazepam; MDZ - midazolam; NZP - nitrazepam; PB - phenobarbital; PentB- Pentobarbital; PHT - phenytoin; PR - propofol; RUF- rufinamide; STP - stiripentol; TP - thiopental; TPM - topiramate; VPA– valproate; LCM-lacosamide; GBP- gabapentin; PG-pregabalin; DZP-diazepam; Ox-CBZ-oxcarbarzepine; PP-perampanel; LD- Loading dose, MD- Maintenance dose; ^£^ Mention of ASMs or anesthetics are not in detail; ^α^ administered only 7 out of 42 cases; ¥ After hospitalization; ^€^ Name of other drugs are not mentioned; ^$^to control emergence reaction; ^#^Mean; ^µ^ Anesthetics were used if seizure were not controlled within 24 h of KE infusion

**Table 3:** Clinical outcomes of super refractory status epilepticus patients treated with ketamine

Author/ Year published	Seizure resolution rate (%)/Resolution time post ketamine	EEG features post-ketamine (%)	Recurrence of SE during hospitalization/ follow up	Functional outcome at discharge	Adverse effects (%)	All-cause mortality (%)
Larger case series						
Gasperd 2013	53.33 (Transient control in an additional 13%)/NR	NR	NR	mRS ≤ 2 (4.3% adults)	PRIS, SVT	43
Sabharwal 2015	91/NR	NR	NR	NR	NR	38.8
Hofler 2016	64/NR	NR	NR	2/7 had no significant disability, 3/7 had severe disability and 2/7 had moderate disability (based of mRS)	NR	59.5 (at 3 years follow up)
Alkhachroum 2020	65/NR	NR	NR	mRS = 5 ± 1 (mean ± SD)	NR	45.6^$^
Smaller case series						
Mewasingh 2003	100/24–48 h	GSW (100)	1/5^#^	Back to usual health (100%)	Irritability (20)	0
Rosati 2012	66.6/ NR	BS (55.55), DTDA (11.11), Transitory BS (1/9)	NR	NR	Hypersalivation (100), transaminitis (44.44)	0
Synowiec 2013	100/4 d– 28 d	NR	NR	Disposition: to home-18%, to LTAC − 27%, to NF- 9%, to IR-27%	NR	18.18.
Basha 2015	73 (36 in case of KE as last drug)	Generalized arciform theta to beta rhythms (7–20 Hz) (45.45)	2/11	Disposition: to home or LTAC- 27.27%, to a NF or IR-45.45%	NR	27.27
Liaqat 2018	40/NR	NR	3/20 had breakthrough or withdrawal seizures	NR	NR	25
Wang 2020	61 (100 in LD and MD receivers) ^¥^ /NR	BS (16.66), DTDA/GSW (4/18)	NR	NR	Hypersalivation (4.44)	22.22
Dericioglu 2020	71 /NR	Suppression of electrographic seizures (22.22), Widespread EEG suppression (14.28)	NR	mRS 5 (4–6) ^*^	Transaminitis (14.28)	28.6
Caranzano 2022	63.7/NR	NR	4/7	New handicapped (6) Complete recovery (1)	NR	36.4
Case reports						
Hsieh 2010	100/5 d	Regular alpha activities resumed in globally attenuated EEG	No SE recurrence	IR	NR	0
Shrestha 2015	100/24–72 h	No seizure activities	NR	NR	NR	50
Mutkule 2018	100/NR	BS	NR	Discharges with no sensory or motor deficit	NR	0
Santoro 2019	100/24–48 h	Decrease in delta brushes (1/3)	No SE recurrence	Complete recovery (1) Persistent neuropsychiatric illness (1)	NR	33
Samanta 2020	100/NR	Diffuse delta slowing with mild suppression (1/2)	½ (in 6 h of KE) ^α^	NR	NR	0
Meenakshi-Sundaram 2020	0	BS ^∞^	Multiple SE recurrence during treatment	NR	NR	100
Manganotti 2021	100/96 h	BS	No SE recurrence	Discharged with no disability	Transaminitis	0

*Abbreviations* mRS-Modified Rankin Scale, SE- status epilepticus, NR-Not reported, PRIS-propofol infusion syndrome, SVT-supraventricular tachycardia, SRSE-Super refractory status epilepticus, h-hours, d- days, BS-Burst suppression, DTDA- Diffuse theta-delta activity, GSW- generalized slow wave, KE-ketamine, LTAC- long term acute care facility, NF-nursing facility, IR-inpatient rehabilitation; ^$^ Mortality in patients with seizure cessation after starting ketamine − 18/31 and mortality in patients without seizure cessation after stopping ketamine − 13/31; ^#^ocurred during 4–9 months follow up and responded to ketamine within 24 h; ^¥^response was 100% in the group which received both loading (LD) and maintenance dose (MD);^*^ median (range); ^α^ seizure free status achieved after second LD and increment in MD; seizure recurred after tapering of KE and Thiopental

**Table 4:** Impact of ketamine on vasopressor requirement

Author/ Year published	Vasopressor use	
	Before KE administration, N	After KE administration, N (Duration)
Gasperd 2013	52	52^&^ (NR)
Sabharwal 2015	53	53^$^ (NR)
Alkhachroum 2020	31	25^#^ (over 5 days)
Synowiec 2013	7	1 (NR)
Liaqat 2018	8	8 (NR)

*Abbreviations* KE-ketamine, ^&^ dose decreased in 6 patients and increased in 21 patients; ^$^ KE and propofol were used in combination; ^#^higher dose of ketamine infusion (OR 1.39, 95% CI 1.38–1.4) and longer administration time (OR 0.9, 95% CI 0.8–1) were associated with a stable mean MAP and a decrease in vasopressor requirements over time (no direct correlation between MAP and ketamine dose)

The corresponding authors of the various studies were contacted via email if the required data were missing, not reported in the manuscript, or reported in an unusual format. In such instances, supplementary materials related with the main paper were also investigated.

## Results

### Search results and study selection

**Fig. 1:**
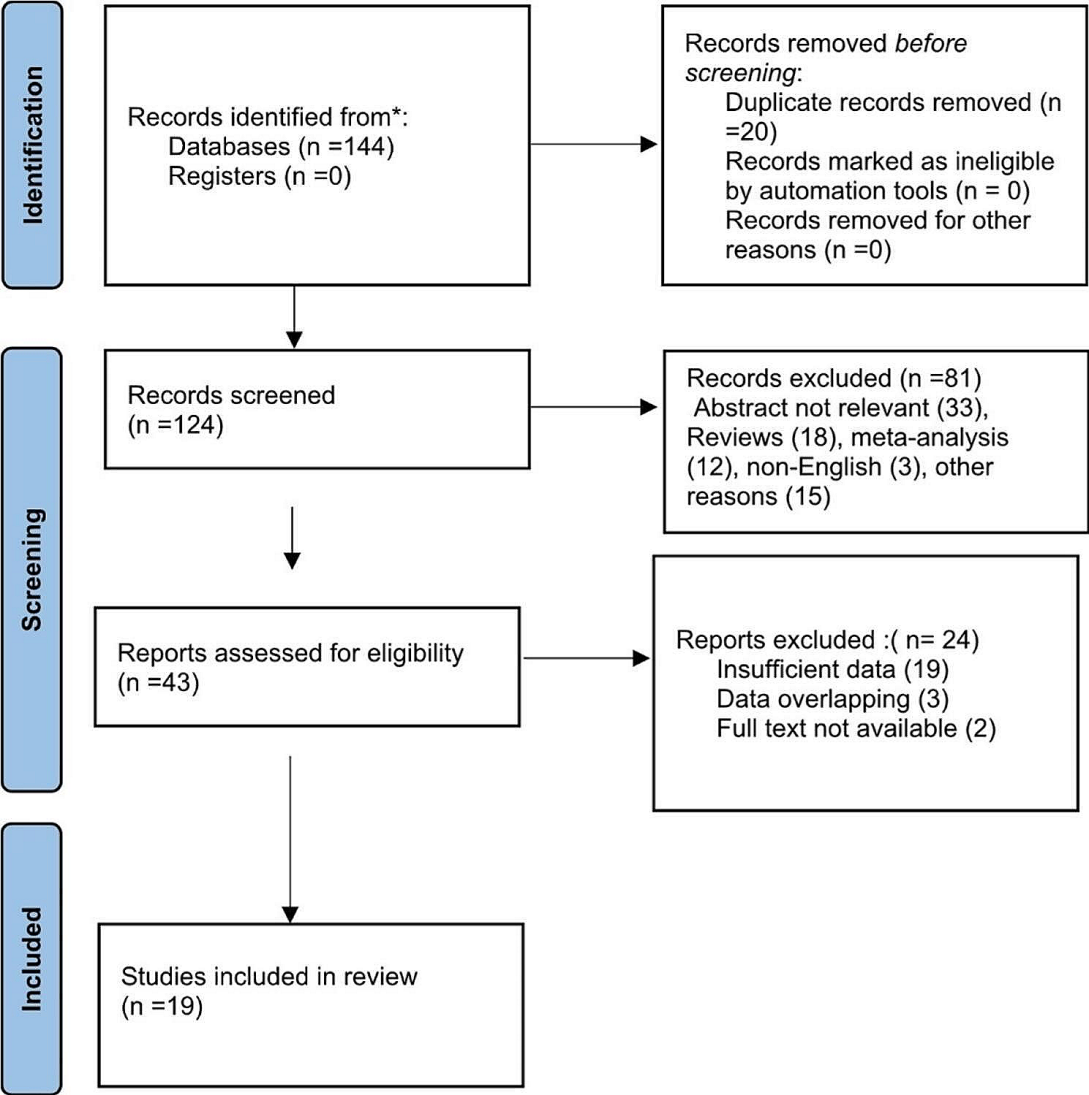
PRISMA flow diagram

We identified 144 studies from electronic database search and no additional studies from manual searching of reference lists and related systematic reviews. After duplicate removal, we screened 124 articles by titles and abstracts. After screening, 43 full-text articles were retrieved and assessed against the predefined inclusion criteria leaving 19 articles eligible to be included in the review. The PRISMA diagram detailing the identification and selection process is given in Fig. 1.

### Baseline demography

There were 336 individuals with 340 cases (episodes) of SE (13 RSE and 327 SRSE) in 19 studies. The age of the participants ranged from 9 months to 86 years. The sample size ranged from 1 to 68 participants. Six studies included pediatric patients, 10 included adult patients and three included both pediatric and adult patients. Geographically, the patients belonged to North America and Europe, USA, Austria, Belgium, Italy, Pakistan, China, Turkey, Switzerland, Taiwan, Nepal, and India. The male to female case ratio was 196:144. For convenience, we have categorized the included studies into larger case series (*N* = 42–68) [13–16], smaller case series (*N* = 5–20) [17–24], and case reports (*N* = 1–3) [25–31] (Table 1).

### Etiology, semiology

#### Etiology

**Fig. 2:**
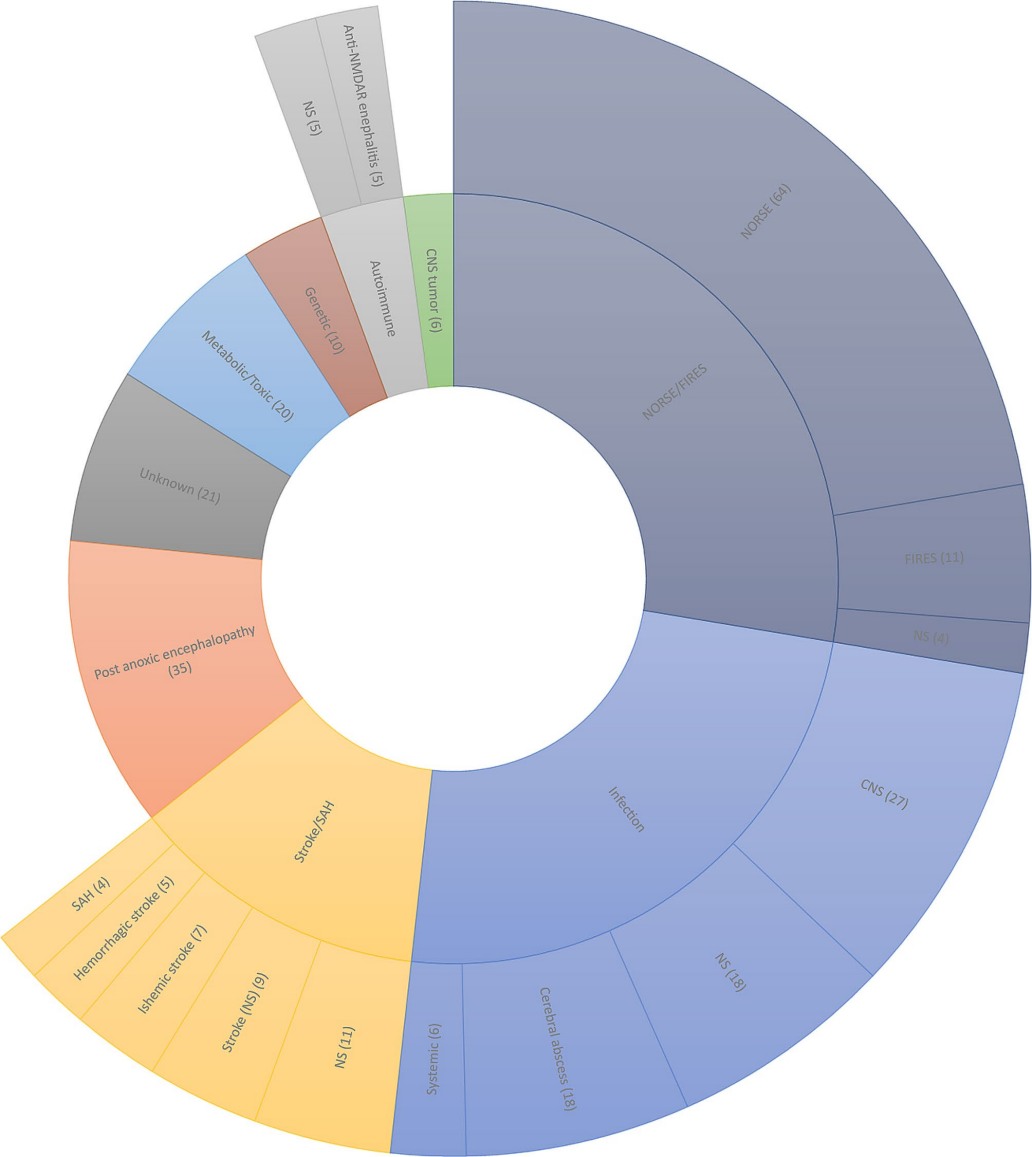
Etiology of SRSE. Abbreviations: NS- not specified. Note-The number of patients presented with particular etiology is given in parentheses; etiology was mentioned for 286 out 336 patients

Most common etiologies identified were new onset RSE (NORSE) / Febrile infection-related epilepsy syndrome (FIRES), followed by infectios (CNS, systemic, and cerebral abscess), stroke/hemorrhages, post anoxic encephalopathy, toxic metabolic, and genetic (Fig. 2). Among adult participants with known cause for SE, infection, anoxia and metabolic cause were the most commonly reported etiology. Infection was the most common etiology in case series of Synowiec et al. and Dericioglu et al. [19, 23, 32]. Anoxia was the most common cause in case series of Hofler et al. and Alkhachroum et al. [15, 16]. Metabolic cause was the most common etiology in case series of Sabharwal et al. followed by anoxia and infection [14]. Gaspard et al. found that more than half of the subjects (57%) were diagnosed as NORSE of unknown etiology, similar to the prospective registry study of Caranzano et al., where the most common etiology was also NORSE/FIRES [13, 24]. Similarly, Liaqat et al. had more than half of the patients (55%) with an unknown etiology of SE [21]. Among pediatric patients, etiologic groups varied widely in case reports and case series, autoimmune and genetic causes were the most common underlying etiology for SE in most pediatric subjects (Table 1).

#### Semiology

The semiology of SE included NCSE (Non-convulsive status epilepticus) and CSE (Convulsive status epilepticus). In a total of 18 studies, there were 197 cases of NCSE and 105 cases of CSE. Semiology was not specified for rest of participants and in the study of Wang et al. In larger case series the NCSE cases hugely outnumbered the CSE cases (Table 1) [13–16].

### Previous treatment received

The drugs used before administration of ketamine included benzodiazepines (BDZ), antiepileptic drugs (ASMs) and anesthetic agents. Benzodiazepines most commonly used as first line therapy included midazolam (MDZ), lorazepam (LZP), diazepam (DZP), clonazepam (CZP) and clobazam (CLB). ASMs included Valproic acid (VPA), Phenytoin (PHT), Fosphenytoin (FPHT), Carbamazepine (CBZ), Levetiracetam (LEV), Topiramate (TPM) and Lacosamide (LCM). Anesthetic agents included Propofol (PR), Thiopental (TP), Phenobarbital (PB) and Pentobarbital (PentB). Besides BZDS, ASMs and anesthetics, other drugs (if used) included steroids and vasopressors. Immunotherapy and Vagal stimulation were other forms of treatment in limited studies (Table 2).

### Latency to ketamine use, form, route of administration and line of therapy

#### Latency to ketamine use

The total duration of SE prior to ketamine administration was highly heterogeneous in such a way that the range varied from a minimum of 6 h to a maximum of 122 days. The median duration ranged from 2 days to 9 days, 4 days to 28 days, and 30 h to 58 days in larger case series (*n* = 42–68), smaller case series (*n* = 5–20), and case reports (*n* = 1–3) respectively (Table 2).

#### Ketamine form and route of administration

In 17 studies, a racemic form of ketamine was used intravenously. Only in case series by Hofler et al. (*N* = 42), S enantiomer of ketamine was used [15]. Only in the case series of Mewasingh et al. (five pediatric NCSE cases ), ketamine was given orally mixed with fruit juice [17].

#### Line of therapy

In 15 studies, ketamine was administered only after the failure of appropriate doses of standard BDZ, ASMs, and conventional anesthetics. In the rest four studies, the chronology of ketamine usage varied among the cases. In the case series of Mewasingh et al., 3 children had oral ketamine as 1st line therapy for their NCSE [17]. Sabharwal et al. described the administration of ketamine and propofol in combination in 67 patients and ketamine was used before propofol in six out of 67 patietns [14]. In case series of Dericioglu et al., except one patient, who received ketamine as the only IV anesthetic due to significant hypotension, ketamine was administered after conventional anesthetic agents [23]. In the case series of Liaqat et al., ketamine was used as first anesthetic agent when midazolam (MDZ), phenytoin (PHT) and levetiracetam (LEV) failed to control seizure [21].

### Ketamine mode of administration, dose and duration

#### Ketamine mode of administration

Administration of ketamine in a bolus dose followed by weight based continuous infusion (maintenance dose) was mentioned in 15 out of 19 studies. Two studies have mentioned about the continuous infusion of ketamine without clarifying about loading dose [16, 24]. Direct infusion was started in all participants of Sabharwal et al. [14]. In cases of Mewasingh et al., oral doses of ketamine was administered twice daily [17].

Hofler et al. had 35 patients who were started with continuous infusion of ketamine without loading dose [15]. Synowiec et al. had one patient on whom no bolus dose was administered [19]. Wang et al. had two groups of patients; seven patients got both loading and maintenance dose of ketamine whereas 11 patients were directly started on maintenance dose [22].

#### Ketamine dose

The loading dose of ketamine ranged from a minimum of 0.5 mg/kg to a maximum of 5 mg/kg. The loading dose of ketamine in pediatric studies ranged from 1 to 3 mg/kg. The maintenance dose ranged from a minimum of 0.05 mg/kg/hour to a maximum of 15 mg/kg/hour. Weight based continuous infusion ranged from 0.5 to 3 mg/kg/h in pediatric studies. The median loading dose and maintenance dose ranged from 1.1 mg/kg to 5 mg/kg and 1.3 mg/kg/hour to 5 mg/kg/hour in the larger and smaller case series (Table 2).

#### Ketamine duration

The duration of ketamine administration ranged from six hours to 30 days. The median duration of larger and smaller case series ranged from 2 days to 8 days. Ketamine administration was withdrawn from 8 patients because of treatment related adverse effects [13, 20, 21, 23].

### Concurrent therapy

Every patient was received one or more concurrent drugs during ketamine infusion in 13 out of 19 studies. In one larger and two smaller case series, 40–86% of participants received concurrent drugs while in two case reports (*N* = 3) there was no concurrent drugs administered with ketamine infusion [15, 21, 23, 27, 29]. The most commonly used concurrent anesthetic agent was midazolam (MDZ) followed by propofol (PR). Concurrent anesthetic other than propofol were thiopental and pentobarbital. Other concurrent drugs were benzodiazepines and ASMs (Table 2**)**. A maximum of 12 concurrent drugs use has been mentioned in the study of Gaspard et al. [13].

### Baseline severity of SE

Severity of status epilepticus was assessed in limited studies. STESS score [33] was used in three case series and a prospective registry study to determine the severity of SE. In the case series by Dericioglu et al. and Alkhachroum et al., favorable STESS (0–2) was present in only one case [16, 23]; in the rest, STESS was unfavorable, falling between 3 and 5. In prospective registry by Caranzano et al., the median STESS score was 3 (range 2–6). There were scarce details about the severity of SE in most studies. The severity of SE (as per the underlying etiology, treatable vs. non-treatable), however, had a definitive role in determining the outcomes as stated in most studies.

### Outcomes

#### Seizure resolution

Seizure resolution rate varied from 53.3 to 91% and 40–100% in larger and smaller case series respectively. Seizure resolution occurred in every case of case reports except in one in which the patient died. Among case series of pediatric age group, resolution rates were 66.7–100%. Wang et al. reported a difference in resolution rates between two groups i.e., 100% in the group receiving both loading and maintenance dose and 36.4% in group receiving only maintenance dose. Duration of ketamine infusion resulting into seizure resolution was mentioned only in two smaller case series and four case reports, which varied from 1 day to 28 days [17, 19, 25, 26, 28, 31] (Table 3).

Six out of seven case reports (with 100% resolution rate) and a case series (with 66.67% resolution rate) consisted only of CSE cases. Two smaller case series consisted only of NCSE cases reported 100% and 71% resolution [17, 23]. In all other studies, patients with both semiology were mixed and no reporting on seizure resolution was done based on semiology. Seizure resolution rate varied from 40 to 100% and 61 to 100% in case series of adult only and pediatric only age group (Table 3).

Resolution rate varied from 40 to 71% in larger and smaller case series with 40–86% concurrent drug users [15, 21, 23]. Two case reports (*N* = 3) reported 100% seizure resolution rate without using any concurrent drugs [27, 29]. The resolution rate varied from 53.3 to 100% in those who received ketamine after failing of conventional BDZ, ASMs, and anesthetics. Resolution rates varied from 40 to 100% in those who received ketamine before failing of all conventional drugs (Tables 2 and 3).

#### EEG features post-ketamine

EEG characteristics after ketamine infusion was reported in 5 small case series and all the case reports (Table 3). Among studies that reported EEG features after ketamine administration, burst suppression pattern was observed in 5/9 children as described by Rosati et al., 3/18 children as described by Wang et al., and in both children of Mutkule et al. and Meenakshi-Sundaram et al. Transitory Burst Suppression pattern was observed in 1/9 children described in case series by Rosati et al. Generalized arciform theta to beta rhythms (7–20 Hz) (5/11), diffuse delta and theta waves (4/18), bilaterally more or less regular alpha activities (approximately 10 Hz) (1/1) and alpha rhythm with sporadic delta activity (1/1) were reported by Basha et al., Wang et al., Hsieh et al. and Manganotti et al., respectively. Diffuse slow activity (5/5), Diffuse theta-delta activity (1/9), Diffuse delta slowing with mild suppression (1/2), and decrease in multi-focal sharp activity (1/3) was observed in studies by Mewasingh et al., Rosati et al., Samanta et al. and Santoro et al. respectively. Though EEG patterns were not reported, suppression or resolutions of ictal activities were assessed as a part of electroclinical seizure cessation in most studies.

#### Recurrence of SE during hospitalization and follow up

Five out of 19 studies viz. Mewasingh et al., Liaqat et al., Caranzano et al., Basha et al. and Meenakshi-Sundaram et al. reported on recurrence of seizures either during hospitalization (10 patients) or during follow up (1 patient) after ketamine treatment [17, 20, 21, 24, 30]. Among ten patients, further treatment with either ketamine or propofol or surgical intervention led to resolution of seizures. However, in the case described by Meenakshi-Sundaram et al., eventually death occurred as result of underlying disease severity.

#### Functional outcome based on mRS

Reporting of functional outcome across all the included studies was highly heterogenous. Only three studies have mentioned the mRS scores. Gaspard et al. reported good functional outcome (mRS ≤ 2) in 2/46 adults further stating no difference in functional outcomes among survivors whether did they respond to ketamine or not [13]. Alkhachroum et al. reported mean mRS score 5 ± 1 on discharge while the baseline mRS was 0 ± 1 [16]. Dericioglu et al. reported a median mRS score 5 (4–6) relating the poor final prognosis to the underlying etiology of NCSE [23]. In the study of Hofler et al., only seven patients had a survival time of more than three years with an outcome according to mRS. 2/7 had no significant disability, 3/7 had severe disability and 2/7 had moderate disability [15]. Caranzano et al. reported new handicapped in six patients, restitution in one while death in four patients among the studied 11 patients [24] (Table 3).

#### Mortality

The reported all-cause mortality varied from 38.8 to 59.5% and 0-36.4% in larger and smaller case series while only one case report (*N* = 1) reported mortality. Gaspard et al. and Hofler et al. showed that no mortality was directly associated with dose and duration of ketamine use. Gaspard et al. also reported that the younger age and response to ketamine was associated with lesser mortality while the increasing age was associated with higher mortality in the study of Hofler et el. Among adults, common causes for mortality included severity of underlying etiology, other medical complications, withdrawal of care per family’s decision or patients’ pre-illness wishes. Other causes included cardiac arrest and brainstem herniation. Among pediatric age group, the cause was not stated in four patients and in the remaining one, the death was due to cardiorespiratory arrest on the background of multiple medical complications.

#### Adverse effects

There were no treatments limiting adverse events following ketamine administration in 15 out of 19 studies (Table 3). The most common adverse effect noted was hypersalivation followed by transaminitis. In 4 studies viz. Gaspard et al., Basha et al., Liaqat et al. and Dericioglu et al., treatment related adverse events led to discontinuation of ketamine in eight subjects. Among the reported ones, one of them developed syndrome similar to Propofol Infusion Syndrome (PRIS) four days after high dose of ketamine and midazolam but no recent propofol use, two patients developed supraventricular tachycardia that resolved after ketamine discontinuation [13]. Ketamine was discontinued in one patient due to a probable adverse event that could not be reliably identified [13]. One patient developed temporary hepatic failure with increased ALT, AST, and GGT after ketamine and these values decreased after withdrawal (he was the oldest patient with the longest duration of ketamine infusion of 24 days) [23]. Other adverse events included end organ damage and severe sepsis but they did not seem to increase in severity after ketamine administration [20]. Liaqat et al. didn’t specify what adverse event led to ketamine withdrawal.

#### Hemodynamic effects

Only five of the included studies have mentioned about the hemodynamic effects (Table 4). Over a duration of five days of ketamine infusion, the number of patients on vasopressor decreased from 31 to 21 in the study of Alkhachroum et al. Synowiec et al. reported weaning of vasopressor in six out of seven patients who were on vasopressor.

## Discussion

RSE and SRSE are dangerous situations requiring swift start of treatment to decrease morbidity and mortality and prevent enduring neurological injury [5]. Low antiseizure medication (ASM) level, traumatic brain injury, intracranial tumor, cerebrovascular disease, autoimmune encephalitis, central nervous system infection, and toxic and metabolic derangements are the common causes [34]. In our review, similar etiologies were responsible for the SRSE and in some patients’ exact etiology was not found (Fig. 2).

RSE and SRSE as the name implies are resistant to commonly used ASMs and the factors that lead to drug resistance in SE include molecular and functional maladaptive changes. With ongoing seizure activity, receptor trafficking occurs due to which intrasynaptic membrane GABA_a_ receptors are internalized and NMDA receptors are upregulated. Hence, though useful in early stages of SE, GABA-ergic drugs such as benzodiazepines and phenobarbital have limited efficacy in prolonged seizure activity. The ideal drug would be the one that is capable of stopping seizures effectively than the current drugs with possible neuroprotective properties to prevent morbidities due to RSE [35, 36]. NMDA receptors are progressively uttered during continued seizure activity which provides a pathophysiological foundation for the use of ketamine in RSE and SRSE due to its NMDA antagonism [35].

A systematic review reported the use of ketamine for RSE with loading doses of 0.5–5 mg/kg and continuous infusion rates of 1–10 mg/kg/h [37]. This systemic review has found similar range of loading and maintenance dose administration (Table 2).

Latency to ketamine administration after onset of seizure seemed to be a pertinent prognostic factor for the efficacy of ketamine. Efficacy of ketamine in the treatment of SE was shown to be maximum when the drug was administered at least one hour after the onset of seizure in an animal model in which the efficacy of ketamine on prolonged seizure was assessed [7]. Ketamine was administered after 24 h in most of the cases of the included studies of this review. Using as a 1st or 2nd line anesthetic agent in the study by Sabharwal et al., the seizure resolution rate was 91% [14]. But, when ketamine was used as a 3rd line anesthetic agent in one case series, seizure resolution rate was found to be only 40%. In the study by Gaspard et al., the subgroup (31% ) receiving ketamine within a median of 4.5 days (6 h to 30 days) after SE onset showed possible or likely response, while there was no response in the rest in which ketamine was administered within a median of 10 days (12 h to 122 days) [13]. Although this leads us to conclude that the administration of ketamine earlier (as a first/second line anesthetic agent) after SRSE diagnosis has better efficacy, this review is not enough for telling precise timing of ketamine administration.

One interesting thing to note is the number of cases of NCSE outnumbered CSE. This is important because there is no consensus with regards to treatment of NCSE as for CSE. The European Federation of Neurological Societies recommends treating NCSE along the same lines as CSE but using non coma inducing drugs before anesthetic agents [38]. Aggressive treatment is warranted in NCSE which follows CSE, acute brain injury, metabolic stress and NORSE as these conditions have poor prognosis [39–41]. Ketamine may prove useful in such circumstances as evidenced by limited data in our review.

Side effects of ketamine consist of both tachy- and bradycardia, hyper- and hypotension, cardiac arrhythmias, hypersalivation, metabolic acidosis, and an emergence phenomenon upon termination [42]. Similar signs were noticed in the patients who developed side effects in the studies included in our review. We identified 8 patients developing few adverse events leading to the discontinuation of ketamine. We found no mortality related directly to the dose and duration of ketamine use. Instead, mortality was found to be positively correlated with age, longer RSE duration, and NCSE in some previous studies [43–45]. Since STESS score is based on patient’s age, level of consciousness, history of seizure, and type of SE, this score will be important in the analysis of mortality in case of ketamine use.

Conventional anesthetics demonstrate an EEG burst suppression while treating SE which represents the goal of treatment along with seizure control [46]. Because of more mixed pattern of EEG observed with the use of ketamine, diffuse slowing (generalized slow wave) and diffuse beta activity should be measured as the marks to attain and hold on same level with burst-suppression pattern [46, 47]. Observation of beta activity, generalized slowing of waves, burst suppression pattern, and several other characteristics were used to assess electroclinical seizure cessation in the studies mentioning EEG changes that were included in our review (Table 3).

Overall, ketamine appeared safe and effective in most of the studies leading to resolution of prolonged seizures. Even if complete resolution was not achieved, ketamine administration led to a significant reduction in seizure burden among patients with SRSE. SRSE with treatable etiology was found to have better outcomes. Also, in most of the studies, early treatment with ketamine was associated with better outcomes of seizure control.

This is by far the most updated and comprehensive review on this topic incorporating all cases. However, there are few limitations of our study. All the included studies are either case reports/series or prospective/retrospective studies and lack a control group, hence the results may not be as accurate. Also, the study design varied in each study resulting in large difference in setting research question, data collection method, and results. The studies included are heterogeneous in terms of timing of administration/dosing/duration/adverse effects of ketamine. There is lack of uniformity in reporting prior and concurrently used drugs, seizure resolution time, and electroclinical outcomes. It is uncertain whether the goal of treatment in SE/RSE/SRSE should be simple cessation of both clinical and electrographic seizures or some degree of suppression of cerebral activity (“burst suppression” or “background suppression/flat line” on EEG). Finally, while we recommend the early introduction of Ketamine in SE/RSE/SRSE, we are not sure of the appropriate timeline when ketamine should be introduced or ketamine should be utilized as 2nd line agent or 3rd line agent. From our review, it is suggested it can be started as early as within 24 h as it led to improved outcomes.

Therefore, it is essential for the future studies to focus on above mentioned aspects such as uniformity on dose/duration/timing of use of ketamine for SE/RSE/SRSE, reporting of prior ans concurrent drugs, uniform electroclinical endpoints and deployment of control group. Also, the future studies should differentiate between NCSE and CSE, pediatric and adult populations in terms of assessment and treatment as they have different prognosis. Not all cases of NCSE require aggressive treatment as evidenced by several studies.

## Conclusion

Ketamine appears to be safe and effective for the management of SRSE, contributing to resolution in many patients and significant reduction in seizure burden in most others. Ketamine is most often attributed to good response when administered early, and mortality rates were not found to be based on ketamine duration or dose, but instead on baseline age and duration of seizures.

### Electronic supplementary material

Below is the link to the electronic supplementary material.

Supplementary Material 1

## Data Availability

Not applicable.

## Declarations

Ethical publication.

We confirm that we have read the Journal’s position on issues involved in ethical publication and affirm that this report is consistent with those guidelines.

## References

[1] Mayer SA, Claassen J, Lokin J, Mendelsohn F, Dennis LJ, Fitzsimmons BF (2002). Refractory status epilepticus: Frequency, risk factors, and impact on outcome. Archives of Neurology.

[2] Holtkamp M, Othman J, Buchheim K, Meierkord H (2005). Predictors and prognosis of refractory status epilepticus treated in a neurological intensive care unit. Journal of Neurology, Neurosurgery and Psychiatry.

[3] Sutter R, Marsch S, Fuhr P, Rüegg S (2013). Mortality and recovery from refractory status epilepticus in the intensive care unit: A 7-year observational study. Epilepsia.

[4] Treiman DM, Meyers PD, Walton NY (1998). A comparison of four treatments for generalized convulsive status epilepticus. Veterans Affairs Status Epilepticus Cooperative Study Group. New England Journal of Medicine.

[5] Trinka E, Cock H, Hesdorffer D (2015). A definition and classification of status epilepticus - report of the ILAE Task Force on classification of Status Epilepticus. Epilepsia.

[6] Holtkamp M (2018). Pharmacotherapy for Refractory and Super-refractory Status Epilepticus in adults. Drugs.

[7] Borris DJ, Bertram EH, Kapur J (2000). Ketamine controls prolonged status epilepticus. Epilepsy Research.

[8] Fujikawa DG (1995). Neuroprotective effect of ketamine administered after Status Epilepticus Onset. Epilepsia.

[9] Prüss H, Holtkamp M (2008). Ketamine successfully terminates malignant status epilepticus. Epilepsy Research.

[10] Shorvon S, Ferlisi M (2011). The treatment of super-refractory status epilepticus: A critical review of available therapies and a clinical treatment protocol. Brain.

[11] Zeiler FA, West M (2015). Ketamine for Status Epilepticus: Canadian physician views and Time to push Forward. Canadian Journal of Neurological Sciences.

[12] Page MJ, McKenzie JE, Bossuyt PM (2021). The PRISMA 2020 statement: An updated guideline for reporting systematic reviews. Bmj.

[13] Gaspard N, Foreman B, Judd LM (2013). Intravenous ketamine for the treatment of refractory status epilepticus: A retrospective multicenter study. Epilepsia.

[14] Sabharwal V, Ramsay E, Martinez R (2015). Propofol-ketamine combination therapy for effective control of super-refractory status epilepticus. Epilepsy & Behavior.

[15] Höfler J, Rohracher A, Kalss G (2016). S)-Ketamine in Refractory and Super-refractory Status Epilepticus: A retrospective study. Cns Drugs.

[16] Alkhachroum A, Der-Nigoghossian CA, Mathews E (2020). Ketamine to treat super-refractory status epilepticus. Neurology.

[17] Mewasingh LD, Seékhara T, Aeby A, Christiaens FJC, Dan B (2003). Oral ketamine in paediatric non-convulsive status epilepticus. Seizure.

[18] Rosati, A., L’erario, M., Ilvento, L. (2012). *Efficacy and Safety of Ketamine in Refractory Status Epilepticus in Children*. 10.1212/WNL.0b013e318278b685 23197747

[19] Synowiec AS, Singh DS, Yenugadhati V, Valeriano JP, Schramke CJ, Kelly KM (2013). Ketamine use in the treatment of refractory status epilepticus. Epilepsy Research.

[20] Basha MM, Alqallaf A, Shah AK (2015). Drug-induced EEG pattern predicts effectiveness of ketamine in treating refractory status epilepticus. Epilepsia.

[21] Liaqat J, Raja W, Wali W, EFFICACY AND SAFETY OF KETAMINE FOR, THE MANAGEMENT OF REFRACTORY STATUS EPILEPTICUS (RSE (2018). IN ADULTS.

[22] Wu J, Wang Q, Qian SY (2020). [Effectiveness of ketamine in the treatment of refractory and super-refractory status epilepticus in children]. Zhonghua Er Ke Za Zhi = Chinese J Pediatr.

[23] Dericioglu, N., Arslan, D., Arsava, E. M., & Topcuoglu, M. A. Efficacy and Safety of Ketamine in refractory / super-refractory Nonconvulsive Status Epilepticus: Single-center experience. Published online 2020. 10.1177/1550059420942677. 10.1177/1550059420942677 32752882

[24] Caranzano, L., Novy, J., & Rossetti, A. O. (2022). Ketamine in adult super- ­ refractory status epilepticus: Efficacy analysis on a prospective registry.;(March):737–742. 10.1111/ane.13610. 10.1111/ane.13610 PMC9310735 35274736

[25] Hsieh CY, Sung PS, Tsai JJ, Huang CW (2010). Terminating prolonged refractory status epilepticus using ketamine. Clinical Neuropharmacology.

[26] Shrestha GS, Joshi P, Chhetri S, Karn R, Acharya SP (2015). Intravenous ketamine for treatment of super-refractory convulsive status epilepticus with septic shock: A report of two cases. Indian J Crit Care Med.

[27] Mutkule D, Rao S, Chaudhuri J, Rajasri K (2018). Successful use of ketamine for burst suppression in super refractory status epilepticus following substance abuse. Indian J Crit Care Med.

[28] Santoro, J. D., Filippakis, A., & Chitnis, T. (2019). Ketamine use in refractory status epilepticus associated with anti-NMDA receptor antibody encephalitis. *Epilepsy Behav Reports*, *12*. 10.1016/j.ebr.2019.100326. 10.1016/j.ebr.2019.100326 PMC6657533 31453565

[29] Samanta D (2020). Ketamine infusion for Super Refractory Status Epilepticus in Alternating Hemiplegia of Childhood. Neuropediatrics.

[30] Meenakshi-Sundaram S, Sankaranarayanan M, Jeyaraman M, Ayyappan C, Karthik SN, Pandi S (2021). Super refractory status in a case of Febrile infection-related Epilepsy Syndrome due to hemophagocytic lymphocytic histiocytosis. Epilepsia Open.

[31] Manganotti P, Cheli M, Dinoto A (2021). Combining perampanel and ketamine in super refractory post-traumatic status epilepticus: A case report. Seizure.

[32] Misra, U. K., Kalita, J., & Dubey, D. (2017). A study of super refractory status epilepticus from India. *Frontiers in Neurology*, *8*(NOV). 10.3389/fneur.2017.00636. 10.3389/fneur.2017.00636 PMC5712310 29234303

[33] Aukland, P., Lando, M., Vilholm, O., Christiansen, E. B., & Beier, C. P. Predictive value of the Status Epilepticus Severity score (STESS) and its components for long-term survival. *BMC Neurol Published Online* 2016:1–9. 10.1186/s12883-016-0730-0. 10.1186/s12883-016-0730-0 PMC5097843 27816063

[34] DeLorenzo RJ, Hauser WA, Towne AR (1996). A prospective, population-based epidemiologic study of status epilepticus in Richmond, Virginia. Neurology.

[35] Wasterlain CG, & Chen JWY (2008). Mechanistic and pharmacologic aspects of status epilepticus and its treatment with new antiepileptic drugs. *Epilepsia*, *49*(SUPPL. 9), 63–73. 10.1111/j.1528-1167.2008.01928.x. 10.1111/j.1528-1167.2008.01928.x 19087119

[36] Feng, H., Mathews, G. C., Kao, C., & Macdonald, R. L. Alterations of GABA A -Receptor function and allosteric modulation during development of Status Epilepticus. *Published Online* 2023:1285–1293. 10.1152/jn.01180.2007. 10.1152/jn.01180.2007 18216225

[37] Rosati A, De Masi S, Guerrini R (2018). Ketamine for Refractory Status Epilepticus: A systematic review. Cns Drugs.

[38] Meierkord H, Boon P, Engelsen B (2010). EFNS guideline on the management of status epilepticus in adults. European Journal of Neurology.

[39] Jirsch J, Hirsch LJ (2007). Nonconvulsive seizures: Developing a rational approach to the diagnosis and management in the critically ill population. Clin Neurophysiol off J Int Fed Clin Neurophysiol.

[40] Vespa PM, Miller C, McArthur D (2007). Nonconvulsive electrographic seizures after traumatic brain injury result in a delayed, prolonged increase in intracranial pressure and metabolic crisis. Critical Care Medicine.

[41] Gaspard N, Foreman BP, Alvarez V (2015). New-onset refractory status epilepticus: Etiology, clinical features, and outcome. Neurology.

[42] Zanos P, Gould TD (2018). Mechanisms of ketamine action as an antidepressant. Molecular Psychiatry.

[43] Ciurans J, Grau-López L, Jiménez M, Fumanal A, Misis M, Becerra JL (2018). Refractory status epilepticus: Impact of baseline comorbidity and usefulness of STESS and EMSE scoring systems in predicting mortality and functional outcome. Seizure.

[44] Madžar D, Geyer A, Knappe RU (2016). Association of seizure duration and outcome in refractory status epilepticus. Journal of Neurology.

[45] Roberg, L. E., Monsson, O., & Kristensen, S. B. (2022). Prediction of long-term Survival after Status Epilepticus using the ACD score.;79(6):604–613. 10.1001/jamaneurol.2022.0609. 10.1001/jamaneurol.2022.0609 PMC9002715 35404392

[46] Rossetti AO, Milligan TA, Vulliémoz S, Michaelides C, Bertschi M, Lee JW (2011). A randomized trial for the treatment of refractory status epilepticus. Neurocritical Care.

[47] Krishnamurthy KB, Drislane FW (1999). Depth of EEG suppression and outcome in barbiturate anesthetic treatment for refractory status epilepticus. Epilepsia.

