# Correction: Use of ketamine in super refractory status epilepticus: a systematic review

**DOI:** 10.1186/s42466-024-00335-2

**Published:** 2024-07-09

**Authors:** Aayush Adhikari, Sushil Kumar Yadav, Gaurav Nepal, Roshan Aryal, Pratik Baral, Peter Neupane, Aadesh Paudel, Barsha Pantha, Sulav Acharya, Gentle Sunder Shrestha, Ramesh Khadayat

**Affiliations:** 1 Manang Hospital, Chame, Manang, 33500 Nepal; 2 Intern, Institute of Medicine, Tribhuvan University Teaching Hospital, Kathmandu, 44600 Nepal; 3 Jibjibe Primary Health Care Centre, Dhaibung, Rasuwa, 45003 Nepal; 4 National Medical College, Birgunj, 44300 Nepal; 5 Post Graduate Institute of Medical Education and Research, Chandigarh, 160012 India; 6 Department of Critical Care Medicine, Tribhuvan University Teaching Hospital, Maharajgunj, Kathmandu, 44600 Nepal

**Correction:** ***Neurol. Res. Pract.*** **6, 33 (2024)**

10.1186/s42466-024-00322-7

Following publication of the original article [[1], the authors reported a typo in the author Gentle Sunder Shrestha’s name.

The name in the original publication was written as “Gentle Sundar Shrestha”.

The correct spelling should read: Gentle Sunder Shrestha

The original article [1] has been updated.
